# In four shallow and mesophotic tropical reef sponges from Guam the microbial community largely depends on host identity

**DOI:** 10.7717/peerj.1936

**Published:** 2016-04-18

**Authors:** Georg Steinert, Michael W. Taylor, Peter Deines, Rachel L. Simister, Nicole J. de Voogd, Michael Hoggard, Peter J. Schupp

**Affiliations:** 1 Institute for Chemistry and Biology of the Marine Environment, Carl von Ossietzky Universität Oldenburg, Wilhelmshaven, Germany; 2 Laboratory of Microbiology, Wageningen University, Wageningen, The Netherlands; 3 School of Biological Sciences, University of Auckland, Auckland, New Zealand; 4 Zoological Institute, Christian-Albrechts-University Kiel, Kiel, Germany; 5 Department of Microbiology and Immunology, University of British Columbia, Vancouver, Canada; 6 Naturalis Biodiversity Center, Leiden, The Netherlands

**Keywords:** 16S rRNA, Microbial diversity, Pyrosequencing, Porifera, Environmental variability, Symbiosis

## Abstract

Sponges (phylum Porifera) are important members of almost all aquatic ecosystems, and are renowned for hosting often dense and diverse microbial communities. While the specificity of the sponge microbiota seems to be closely related to host phylogeny, the environmental factors that could shape differences within local sponge-specific communities remain less understood. On tropical coral reefs, sponge habitats can span from shallow areas to deeper, mesophotic sites. These habitats differ in terms of environmental factors such as light, temperature, and food availability, as well as anthropogenic impact. In order to study the host specificity and potential influence of varying habitats on the sponge microbiota within a local area, four tropical reef sponges, *Rhabdastrella globostellata*, *Callyspongia* sp., *Rhaphoxya* sp., and *Acanthella cavernosa*, were collected from exposed shallow reef slopes and a deep reef drop-off. Based on 16S rRNA gene pyrosequencing profiles, beta diversity analyses revealed that each sponge species possessed a specific microbiota that was significantly different to those of the other species and exhibited attributes that are characteristic of high- and/or low-microbial-abundance sponges. These findings emphasize the influence of host identity on the associated microbiota. Dominant sponge- and seawater-associated bacterial phyla were Chloroflexi, Cyanobacteria, and Proteobacteria. Comparison of individual sponge taxa and seawater samples between shallow and deep reef sites revealed no significant variation in alpha diversity estimates, while differences in microbial beta diversity (variation in community composition) were significant for *Callyspongia* sp. sponges and seawater samples. Overall, the sponge-associated microbiota is significantly shaped by host identity across all samples, while the effect of habitat differentiation seems to be less predominant in tropical reef sponges.

## Introduction

In marine ecosystems, sponges represent common and versatile members of the benthos, with distribution ranges along large environmental gradients and across various habitats including deep sea benthos, seamounts, polar regions, and temperate and tropical coral reefs (Bell, 2008). Many sponges are notable for their diverse and abundant microbial biota, with up to 35% of sponge biomass being made up of microbes (Taylor et al., 2007). Sponge-microbe relationships can include microbial cells as a food source for filter-feeding sponges, carbon- and nitrogen-based nutritional interactions, and the synthesis of secondary metabolites for chemical defence mechanisms (Hentschel et al., 2012; Taylor et al., 2007). Accumulated evidence indicates that much of the sponge microbiota is specific to, or at the very least heavily enriched in, sponge hosts (Lee et al., 2011; Pita et al., 2013; Schmitt, Hentschel & Taylor, 2012; Webster et al., 2010; Simister et al., 2012a). Even studies which detect so-called “sponge-specific” microbes outside the sponge host only find these at very low abundances, with no evidence for these free-living microbes being metabolically active (Taylor et al., 2013; Moitinho-Silva et al., 2014). In addition to the apparent influence of host identity on microbial composition (Easson & Thacker, 2014; Naim et al., 2014; Pita et al., 2013; Reveillaud et al., 2014), marine sponge-associated microbial communities exhibit relatively high temporal and biogeographic stability (e.g., Simister et al., 2013; Hardoim & Costa, 2014; Taylor et al., 2005).

While sponges occupy a range of different depths, knowledge about the influence of depth on the composition of the sponge microbiota still remains rather limited (Olson & Kellogg, 2010; Olson & Gao, 2013; Morrow, Fiore & Lesser, 2016). Spatial dynamics of the host-associated microbiota within coral reef ecosystems, from shallow (0–30 m) to mesophotic (30–150 m) sites, are of great interest because of the potential role of the mesophotic coral ecosystem (MCE) as refugia for both microbial symbionts and their hosts facing threats of environmental change and anthropogenic disturbances (Olson & Kellogg, 2010; Lesser, Slattery & Leichter, 2009; Kahng, Copus & Wagner, 2014). Sponges in particular seem to be very important benthic members of MCEs, with increased growth rates, biomass and coverage compared to their shallow site counterparts (Lesser, Slattery & Leichter, 2009). The habitat of sponges can span from shallow reef ecosystems into these mesophotic zones, which are less influenced by variable abiotic factors such as surface water temperature and salinity, or by direct human impact such as overfishing and pollution (Kahng, Copus & Wagner, 2014; Olson & Kellogg, 2010). Research on thermal stress responses of sponge-associated microbial communities, for example, has already shown the drastic effects of rising water temperatures on the microbial symbionts (Webster, Cobb & Negri, 2008; Simister et al., 2012b). Knowledge of the spatial dynamics of potential microbial refugia could yield new perspectives on the resilience and management of coral ecosystems, which are facing enormous pressures due to increasing global climatic disturbances and anthropogenic influences along highly populated and narrow land-sea transition zones (Olson & Kellogg, 2010; Ainsworth, Thurber & Gates, 2010).

It has been suggested that inter-habitat connectivity of the host-associated microbial biota between the light flooded subsurface and the twilight areas of the MCEs exists because of larval migration, water circulation and the filtering activities of sessile benthic invertebrates that inhabit these zones (Olson & Kellogg, 2010; Slattery et al., 2011; Kahng, Copus & Wagner, 2014; Thacker & Freeman, 2012). The first assessment of *in situ* sponge-associated communities along an MCE depth gradient suggested host-specific local variations in community structure, which are possibly influenced by prevailing biotic and abiotic factors (Olson & Gao, 2013). A recent study of the *Xestospongia muta* microbiota with parallel inorganic nutrient and stable isotope analyses demonstrated that changing environmental factors with depth contribute to the microbial 16S rRNA gene-defined microbial compositions in this sponge (Morrow, Fiore & Lesser, 2016).

In this study, we apply high-throughput 16S rRNA gene amplicon pyrosequencing to profile four demosponge species*, Rhabdastrella globostellata*, *Callyspongia* sp., *Acanthella cavernosa*, and *Rhaphoxya* sp., collected with surrounding seawater to address the following aims: (1) based on all available samples we investigate the degree of host specificity of microbial communities among several tropical sponge species, and (2) based on a habitat-specific subset of samples we estimate the influence of local habitat variation on sponge- and seawater-associated microbial community patterns. We sampled two closely related but environmentally differentiating habitats; a deep drop-off (Guam Blue Hole) for the collection of the mesophotic reef samples and nearby shallow reef slope sites for comparison.

**Table 1:** Sample data. With internal sample name, classified taxon, date and site of collection, depth and coordinates for each sample.

Acc	Sample type	Site	Depth (m)	Date	Coordinates
A1	*A. cavernosa*	Near Blue Hole	5	29-Jun-10	N13.26.180; E144.37.504
A2	*A. cavernosa*	Tanguisson	77	04-Nov-10	N13.32.620; E144.48.265
A3	*A. cavernosa*	Hospital Point	92	17-Mar-08	N13.30.126; E144.46.092
C1	*Callyspongia* sp.	Gab Gab	4.5	06-Jul-10	N13.26.35; E144.38.36
C2	*Callyspongia* sp.	Western Shoals	4.5	06-Jul-10	N13.27.018; E144.39.120
C3	*Callyspongia* sp.	Gab Gab	4.5	06-Jul-10	N13.26.35; E144.38.36
C4	*Callyspongia* sp.	Blue Hole	80–90	23-Jan-08	N13.26.180; E144.37.504
C5	*Callyspongia* sp.	Blue Hole	80–90	23-Jan-08	N13.26.180; E144.37.504
C6	*Callyspongia* sp.	Blue Hole	88	29-Jun-10	N13.26.180; E144.37.504
C7	*Callyspongia* sp.	Blue Hole	80–90	06-Jul-10	N13.26.180; E144.37.504
RG1	*R. globostellata*	Blue Hole	80–90	29-Jun-10	N13.26.180; E144.37.504
RG2	*R. globostellata*	Blue Hole	80–90	29-Jun-10	N13.26.180; E144.37.504
RG3	*R. globostellata*	Blue Hole	80–90	29-Jun-10	N13.26.180; E144.37.504
RG4	*R. globostellata*	Western Shoals	1.5-3	25-Jun-10	N13.27.018; E144.39.120
RG5	*R. globostellata*	Western Shoals	1.5-3	25-Jun-10	N13.27.018; E144.39.120
RG6	*R. globostellata*	Western Shoals	1.5–3	25-Jun-10	N13.27.018; E144.39.120
RS1	*Rhaphoxya* sp.	Blue Hole	95	06-Jul-10	N13.26.180; E144.37.504
RS2	*Rhaphoxya* sp.	Blue Hole	80–90	06-Jul-10	N13.26.180; E144.37.504
RS3	*Rhaphoxya* sp.	Blue Hole	80–90	06-Jul-10	N13.26.180; E144.37.504
RS4	*Rhaphoxya* sp.	Blue Hole	80-90	06-Jul-10	N13.26.180; E144.37.504
RS5	*Rhaphoxya* sp.	Blue Hole	77	29-Jun-10	N13.26.180; E144.37.504
W1	Waterfilter	Blue Hole	88	06-Jul-10	N13.26.180; E144.37.504
W2	Waterfilter	Blue Hole	88	06-Jul-10	N13.26.180; E144.37.504
W3	Waterfilter	Blue Hole	88	06-Jul-10	N13.26.180; E144.37.504
W4	Waterfilter	Western Shoals	3	06-Jul-10	N13.27.018; E144.39.120
W5	Waterfilter	Western Shoals	3	06-Jul-10	N13.27.018; E144.39.120

## Materials & Methods

### Sample processing and sequencing

Samples of four different sponge species *R. globostellata* (*n* = 6 specimens), *Callyspongia* sp. (*n* = 7), *A*. *cavernosa* (*n* = 3), *Rhaphoxya* sp. (*n* = 5) and seawater (*n* = 5) were collected from closely connected Guam reef sites and depths (shallow exposed reef sites and a deep drop-off) via snorkelling and technical diving (Table 1). The tropical island Guam is known for the presence of a marine karstic limestone sinkhole (i.e., Guam Blue Hole); Taborosi, Jenson & Mylroie (2003). The vertical Blue Hole sinkhole has a vertical shaft of more than 90 m depth at which it merges with a deep drop-off running along the southern part of the Orote peninsula. This site harbours a rich and diverse associated coral reef fauna (Paulay, 2003) with very different environmental conditions from the shallow coral slopes of the surrounding areas. All shallow water samples for *R. globostellata* and *Callyspongia* sp. were collected from either Western Shoals or Gab Gab, which are both inside Apra Harbor and separated less than 1 nautical mile. The samples were collected in the same habitat, a shallow fore reef slope, which is dominated by *Porites rus* corals. Sample collection took place between June 25th 2010 and July 6th 2010, except for the samples C4 & C5 (March 2010) and A2 & A3 (November 2010 and March 2008, respectively) (see Table 1 for the sample accession codes). Sampling was carried out by technical diving, using trimix of helium, nitrogen and oxygen with mixes varying between target depths (oxygen 10–15%, helium 25–45%, nitrogen making up the balance). Decompression stops were carried out with nitrox mixes of 32–40%, which was switched to 75–82% oxygen once a decompression depth of 9 m was reached. Due to the various gas mixes, dive equipment included 5 independent tanks. Given that bottom times were limited to ca. 10–15 min at 90 m including descent time, searching for replicate sponges was very limited, explaining the somewhat limited number of replicates for the deep drop-off sponges. Sea surface temperatures (SST) were obtained from the Coastal Data Information Program (CDIP, http://cdip.ucsd.edu/themes/). SST averaged 29.8 °C in June and 29.7 °C in July 2010. Temperatures at depth (90 m) averaged 25 °C (taken with Dive rite-Intel HE and VR3-trimix dive computers). All samples were frozen, freeze-dried, and then stored at −20 °C prior to further processing. Sponges were identified by Dr Nicole J. de Voogd and vouchers of each species were preserved in 70% ethanol at the Naturalis Biodiversity Center, Leiden, Netherlands. Genomic DNA was extracted from sponge tissue and water filters (1 L each, 0.22 µm filter) by bead-beating in an ammonium acetate buffer, as previously described (Taylor et al., 2004). 16S rRNA gene amplification with primers targeting the V4-V5 region (454MID_533F: GTGCCAGCAGCYGCGGTMA and 454_907RC: CCGTCAATTMMYTTGAGTTT) and purification for pyrosequencing was performed as previously described by Simister et al. (2012b). Pyrosequencing was performed by Macrogen Inc. (Seoul, South Korea) using the Roche GS FLX Titanium system. The obtained raw sequence data can be accessed via the NCBI Sequence Read Archive under accession number SRX838037.

### Raw sequence processing

Sequences were processed using mothur v.1.33.0 (Schloss, Gevers & Westcott, 2011; Schloss et al., 2009). Pyrosequencing flowgrams were filtered and denoised using the mothur implementation of AmpliconNoise (Quince et al., 2011). Adaptor, MID, and primer sequences were removed from raw sequences. Sequences were removed from the analysis if they were ≤200 bp or contained ambiguous characters, homopolymers longer than 8 bp, more than one MID mismatch, or more than two mismatches to the reverse primer sequence. Unique sequences were aligned against a SILVA alignment (available at http://www.mothur.org/wiki/Silva_reference_alignment). After chimera-checking with UCHIME (Edgar et al., 2011), unique sequences were identified using the Greengenes “gg_13_8_99” reference taxonomy (available at http://www.mothur.org/wiki/Greengenes-formatted_databases). Non-target sequences (e.g. chloroplasts, mitochondria, eukaryotic 18S rRNA) were removed.

### Sequence data analyses

After raw data processing, mothur was used to group the obtained high quality sequences into 97% average neighbour sequence-similarity threshold operational taxonomic units (i.e., 97%-OTUs), for calculation of Shannon & inverse Simpson diversity, and rarefaction curves. Reads were evenly subsampled to 2,387 sequences per sample for all alpha diversity calculations (mothur command *summary.single* & *subsample*=*T*; 1,000 iterations). For visualization and interpretation of the microbial community data, we used standardized 97%-OTU abundance information (vegan command *decostand* & *method*= *hellinger* or *pa*; Bray-Curtis dissimilarities for relative abundance & Jaccard dissimilarities for presence/absence analyses). To estimate the variance of beta diversity, two hypothetical treatments were applied to the dataset: (a) ‘habitat’ (shallow reef slope, deep drop-off) and (b) ‘group’ (*Rhabdastrella*, *Rhaphoxya*, *Callyspongia*, *Acanthella*, seawater) (Table S1). These treatments were used for analysis of multivariate homogeneity of group dispersions (variances) (Anderson, 2006) with the *betadisper* (followed by pairwise Tukey’s Honestly Significant Difference tests) and *permutest* function from the vegan package in R (v. 3.0.2) (Oksanen et al., 2012; R Development Core Team, 2013). We used the *adonis* function (1000 permutations) from the vegan package to estimate the variances in beta diversity for both treatment groups (Anderson, 2001). Visualization of variations in sponge composition among habitats (i.e., shallow reef slope, deep drop-off) and sponge hosts (*R. globostellata*, *Callyspongia* sp., *Rhaphoxya* sp. and *A. cavernosa*) were assessed with multivariate non-metric multidimensional scaling (nMDS) using the *metaMDS* function from the vegan package. Hypothesis-based treatments were added as dispersion ellipses to the ordination plots with the vegan function *ordieellipse* (0.95 confidence interval). All multivariate analyses were performed with relative abundance and presence/absence data. The contribution of OTUs to average overall pairwise sample dissimilarity in *R. globostellata*, *Callyspongia* sp. and seawater specific datasets (‘habitat’ treatment) was assessed using the vegan function *simper* for similarity percentages (SIMPER). Relative abundance of the 30 most abundant OTUs (each ≥0.5% relative abundance) and relative phylum abundance were visualized with JColorGrid (Joachimiak, Weisman & May, 2006). Representative sequences of the 30 most abundant OTUs were assembled via the *get.oturep* command in mothur and BLAST searched against the NCBI Nucleotide collection (discontiguous megablast & Models excluded). The best hits and representative sequences for the most abundant OTUs can be accessed via the Figshare online repository (https://dx.doi.org/10.6084/m9.figshare.2366827). Hierarchical clustering based on all OTUs was performed using the vegan package in R via the function *vegdist* (Bray-Curtis dissimilarity) and *hclust* (*method* = *average*) and subsequently added onto the phylum fingerprint. As described above, all samples were included in the group-based (i.e., sponge and seawater samples) multivariate community (adonis and betadisper) and ordination (nMDS) analysis. In the subsequent habitat-based (shallow reef slope vs. deep drop-off) comparison, *A. cavernosa*and *Rhaphoxya* sp. samples were omitted from all analyses, and seawater samples from multivariate community analysis and ordination analyses, due to insufficient numbers of replicates in the dataset for the particular habitats. The 97% OTU abundance table combined with the Greengenes classification for each individual OTU can be accessed via the Figshare online repository (https://dx.doi.org/10.6084/m9.figshare.2063280).

## Results

**Figure 1:**
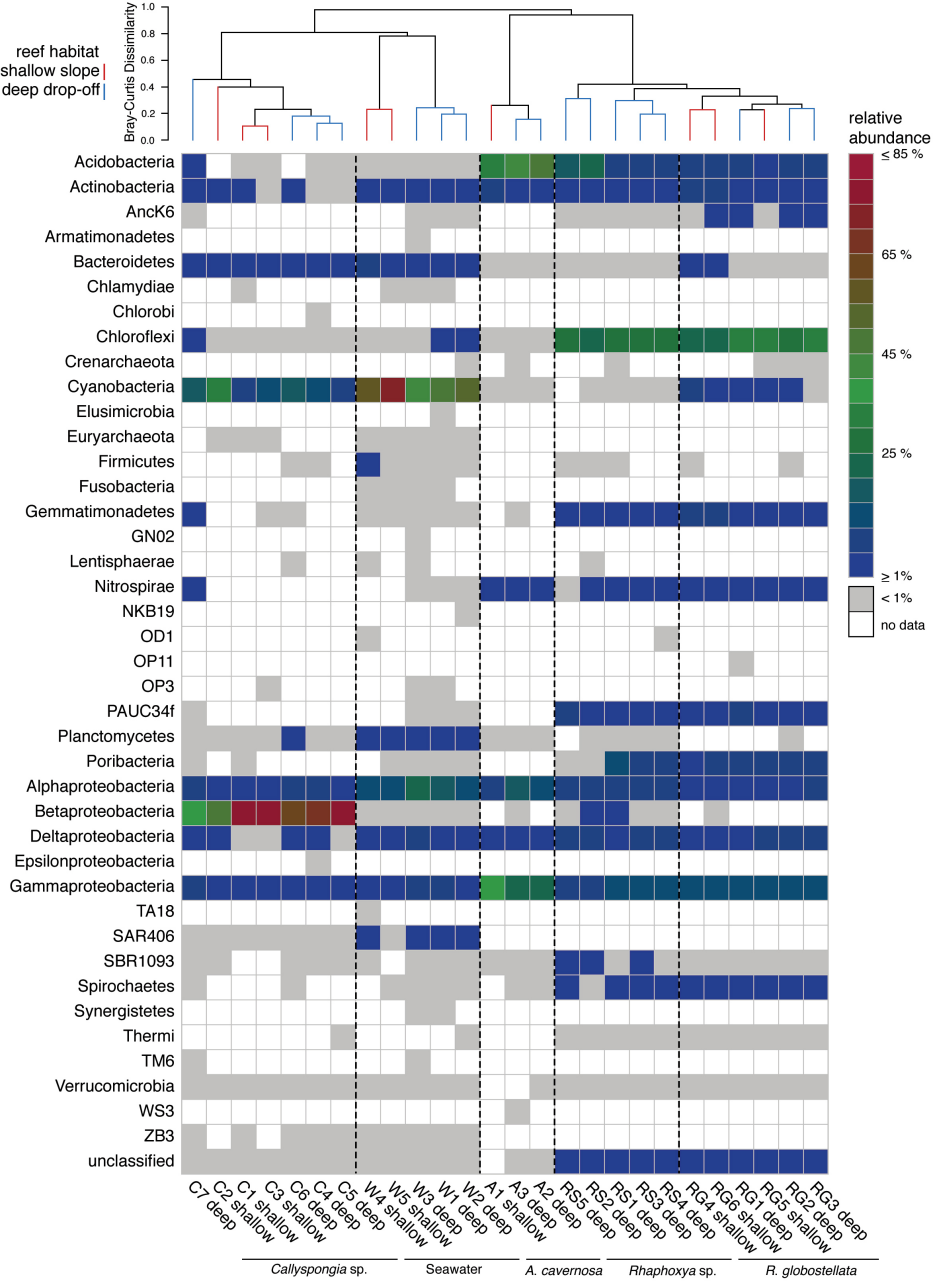
Phylum abundance and distribution. Heatmap of the relative abundance of 16S rRNA gene amplicon sequences taxonomically classified to phylum level. The dendrogram is based on Bray-Curtis dissimilarities (relative abundances of 97%-OTUs).

In total, 191,710 sequences were retained after denoising and quality control. Between all sampling groups (four sponge taxa and one seawater group), number of sequences, observed and average 97%-OTUs were higher overall within the seawater group in comparison to the sponge groups (Table 2). Coverage was slightly higher for *A. cavernosa* and *R. globostellata* compared to seawater and *Callyspongia* sp. samples (Table 2). These group-specific observations were also reflected in the rarefaction curves (Fig. S1). Across 26 samples (21 sponges and 5 seawater samples), 2247 OTUs (97% cut-off) were determined. After Greengenes classification, these OTUs were assigned to 33 bacterial and two archaeal phyla (Fig. 1). Pooled seawater samples showed the highest phylum richness, with 30 bacterial and two archaeal phyla identified. The observed phylum-level diversity of *R. globostellata* and *Rhaphoxya* sp. was very similar with 18 and 19 bacterial phyla, respectively, and Crenarchaeota as the archaeal phylum present in both sponge species. The main differences in bacterial composition between *Rhaphoxya* sp. and *R. globostellata*were in the relative abundances of Cyanobacteria and Betaproteobacteria (Fig. 1). However, compared to the other three groups, these two sponges were more similar to each other at microbial phylum level. In contrast, *Callyspongia* sp. exhibited an association with Euryarchaeota and 24 bacterial phyla. *A. cavernosa* deviated slightly from the so far observed phylum richness, with only 13 bacterial phyla and Crenarchaeota (Fig. 1). Almost half of the occurring phyla were present in all five groups (e.g., Acidobacteria, Actinobacteria, Bacteroidetes, Chloroflexi, Cyanobacteria, Nitrospirae, Planctomycetes, Proteobacteria, Spirochaetes, Synergistetes, and Verrucomicrobia), but exhibited group-specific variation (Fig. 1). Hierarchical clustering based on Bray-Curtis dissimilarity distances revealed group-specific clades with high between-group and low within-group dissimilarities (Fig. 1 & Fig. S2A). In addition, individual Bray-Curtis dissimilarity clustering of all five groups separated almost every sample in accordance to the sampled habitat (Figs. S2B–S2E), while subsequent multivariate analyses only confirmed significant differences between habitats for *Callyspongia* sp. and seawater samples (Table 3).

**Table 2:** Sample sequence statistics. Sequence and OTU of 97%-OTUs (subsampling size based on the sample with the fewest sequences emphasized in bold = 2,387 reads). Subsequent coverage, richness, and alpha diversity estimates are based on the subsampled dataset.

Acc	Sample	Reef habitat	Total OTUs	# sequences	Average OTUs	Coverage	Invsimpson	Shannon
A1	*A. cavernosa*	Shallow slope	56	5,500	40	0.99	6.04 ± 0.69	2.32 ± 0.1
A2	*A. cavernosa*	Deep drop-off	78	7,076	50	0.99	4.04 ± 0.54	2.12 ± 0.12
A3	*A. cavernosa*	Deep drop-off	86	6,351	55	0.99	5.61 ± 0.81	2.4 ± 0.11
C1	*Callyspongia* sp.	Shallow slope	131	5,530	87	0.98	1.56 ± 0.12	1.13 ± 0.16
C2	*Callyspongia* sp.	Shallow slope	132	3,474	114	0.98	3.12 ± 0.29	1.94 ± 0.16
C3	*Callyspongia* sp.	Shallow slope	170	4,361	114	0.97	1.63 ± 0.13	1.14 ± 0.16
C4	*Callyspongia* sp.	Deep drop-off	165	5,077	117	0.98	2.02 ± 0.2	1.58 ± 0.17
C5	*Callyspongia* sp.	Deep drop-off	188	7,322	105	0.98	1.56 ± 0.12	1.1 ± 0.16
C6	*Callyspongia* sp.	Deep drop-off	133	**2,387**	133	0.97	2.36 ± 0.24	1.74 ± 0.17
C7	*Callyspongia* sp.	Deep drop-off	321	4,433	258	0.96	5.45 ± 0.84	3.2 ± 0.19
RG1	*R. globostellata*	Deep drop-off	206	4,917	181	0.99	62.86 ± 8.51	4.53 ± 0.08
RG2	*R. globostellata*	Deep drop-off	198	4,423	173	0.98	63.06 ± 7.14	4.47 ± 0.08
RG3	*R. globostellata*	Deep drop-off	199	3,635	183	0.98	66.03 ± 8.09	4.54 ± 0.08
RG4	*R. globostellata*	Shallow slope	205	9,488	167	0.99	37 ± 7.31	4.31 ± 0.1
RG5	*R. globostellata*	Shallow slope	206	5,803	181	0.99	64.79 ± 8.73	4.55 ± 0.08
RG6	*R. globostellata*	Shallow slope	230	10,773	177	0.99	57.45 ± 8.95	4.49 ± 0.09
RS1	*Rhaphoxya* sp.	Deep drop-off	289	6,262	214	0.97	43.3 ± 8.24	4.47 ± 0.11
RS2	*Rhaphoxya* sp.	Deep drop-off	228	7,092	172	0.98	51.53 ± 7.47	4.39 ± 0.09
RS3	*Rhaphoxya* sp.	Deep drop-off	205	4,175	181	0.98	60.78 ± 9.04	4.52 ± 0.08
RS4	*Rhaphoxya* sp.	Deep drop-off	222	7,539	177	0.99	62.81 ± 8.27	4.52 ± 0.08
RS5	*Rhaphoxya* sp.	Deep drop-off	217	9,372	169	0.99	56.58 ± 7.89	4.44 ± 0.08
W1	Waterfilter	Deep drop-off	555	11,606	276	0.94	5.09 ± 0.86	3.27 ± 0.2
W2	Waterfilter	Deep drop-off	592	7,740	334	0.92	4.18 ± 0.66	3.18 ± 0.21
W3	Waterfilter	Deep drop-off	782	18,007	345	0.93	6.94 ± 1.35	3.74 ± 0.19
W4	Waterfilter	Shallow slope	493	19,586	174	0.96	4.52 ± 0.72	2.83 ± 0.17
W5	Waterfilter	Shallow slope	342	9,781	160	0.96	2.53 ± 0.3	2.15 ± 0.19

**Figure 2:**
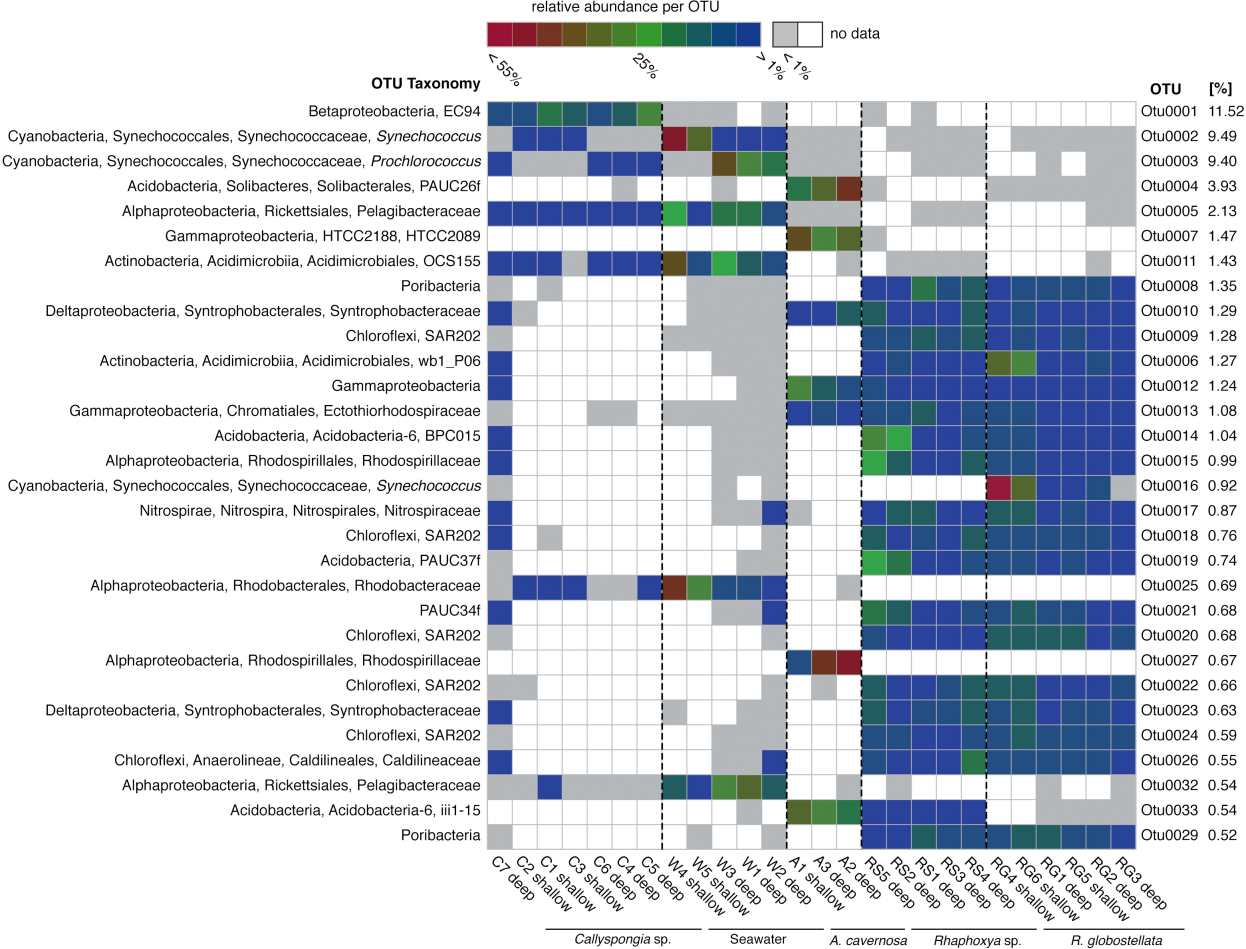
Most abundant 97%-OTUs. Fingerprint of the 30 most abundant 97%-OTUs (>0.5%) based on relative abundance for each individual OTU. Greengenes classifications from phylum to species level are provided on the left. On the right are relative abundances of the individual 97%-OTUs in relation to all detected OTUs.

**Table 3:** Multivariate analyses. Analysis of Bray-Curtis (relative abundance) and Jaccard (presence-absence) dissimilarities among all samples (group based) and the *R. globostellata*, *Callyspongia* sp., and seawater specific subsets (habitat based). Results represent the three groups and ordination ellipses from Figs. 3A–3C and seawater with the number of 97%-OTUs available in each individual dataset for the betadisper/permutest (Dispersion) and adonis (PERMANOVA) analyses (1,000 permutations each). Attached are the nMDS stress values of the multivariate ordination from Figs. 3A–3C and seawater (seawater ordination not shown). Significant differences in bold.

Data matrix	Sample groups		Dispersion			PERMANOVA			
		OTUs	df	*F* value	*p* value	*F* value	*R*^2^	*p* value	nMDS stress
Relative abundance	Sponges & water (groups)	2,247	21	1.75	>0.99	22.09	0.81	<**0.001**	0.08
	*R. globostellata* (habitat)	341	4	0.01	>0.99	1.80	0.31	0.1	0.01
	*Callyspongia* sp. (habitat)	673	5	0.29	>0.99	2.85	0.36	<**0.001**	<0.01
	Seawater (habitat)	1,537	3	5.23	**0.02**	6.28	0.68	**0.008**	<0.01
Presence-absence	Sponges & water (groups)	2,247	21	31.38	**0.02**	6.15	0.54	<**0.001**	0.09
	*R. globostellata* (habitat)	341	4	0.50	>0.99	1.64	0.290	0.1	<0.01
	*Callyspongia* sp. (habitat)	673	5	0.26	>0.99	1.85	0.270	<**0.001**	<0.01
	Seawater (habitat)	1537	3	0.62	>0.99	2.4	0.45	0.1	<0.01

**Figure 3:**
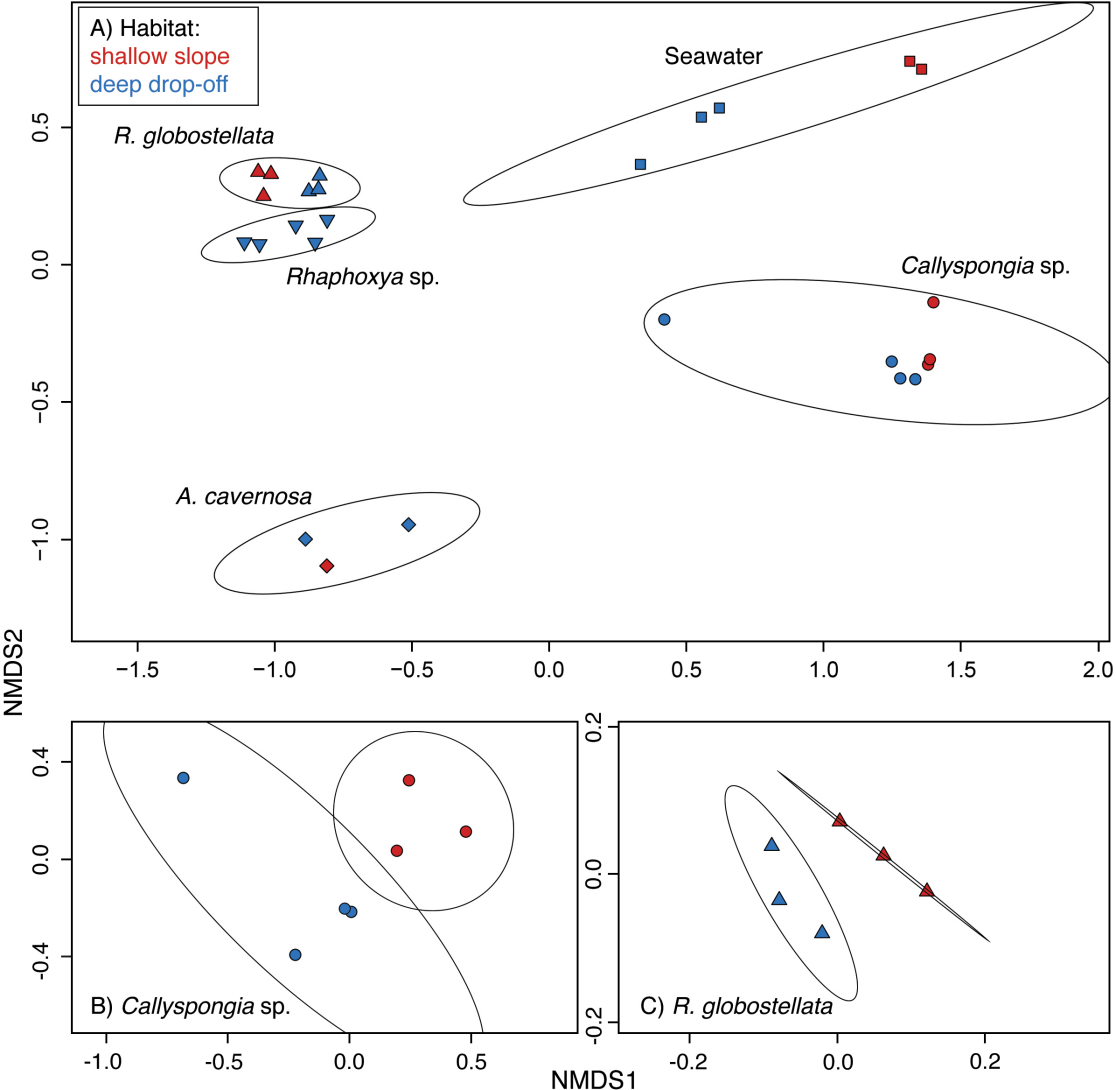
97%-OTU community structure. nMDS ordinations based on Bray-Curtis (relative abundance) dissimilarities (97%-OTUs). (A) all samples and ordination ellipse for each sample group, (B) only *Callyspongia* sp. with ordination ellipse for each habitat, and (C) *R. globostellata* with ordination ellipse for each habitat.

The most abundant OTUs (*n* = 30) included members of the Proteobacteria (Alpha-, Beta-, Delta-, and Gamma-), Cyanobacteria, Acidobacteria, Chloroflexi, “Poribacteria”, Actinobacteria, Nitrospirae and PAUC34f (Fig. 2). The distribution of the three most abundant OTUs (OTU0001: Betaproteobacteria, uncultured order EC94; OTU0002: Cyanobacteria, *Synechococcus*; OTU0003: Cyanobacteria, *Prochlorococcus*) was mostly limited to *Callyspongia* sp. and seawater samples. Among the abundant *R. globostellata* OTUs were representatives of Chloroflexi class SAR202, the candidate phylum “Poribacteria,” various Acidobacteria and Proteobacteria, and the uncultured sponge symbiont PAUC34f. Interestingly, while most abundant OTUs in *R. globostellata* and *Rhaphoxya* sp. were largely shared, and evenly distributed overall, the abundant OTU0016 (*Synechococcus*) in *R. globostellata* was not detected in *Rhaphoxya* sp. On the contrary, the latter sponge species featured an Acidobacteria OTU (OTU0033) that was predominantly shared with *A. cavernosa* instead of *R. globostellata* (Fig. 2). Additionally, three OTUs were highly abundant only in *A*. *cavernosa*: OTU0004 (Acidobacteria, PAUC26f), OTU0007 (Gammaproteobacteria, HTCC2089), and OTU0027 (Alphaproteobacteria, Rhodobacteraceae) (Fig. 2).

Non-metric multidimensional scaling plots created with all samples showed a high degree of sample-specific pooling (Fig. 3A). The variation among sample-specific groups was significant for the relative abundance and presence-absence datasets (Table 3). Pairwise comparisons of mean group dispersions revealed the significant contributions of seawater and *Callyspongia* sp. samples to the differences between the groups (Tables S2 and S3).The habitat-based multivariate analysis of variance was significant for *Callyspongia* sp. and seawater samples (Table 3). The nMDS ordination showed apparent habitat-related community clusters for both (*Callyspongia* sp. and *R. globostellata*) sponge taxa (Figs. 3B and 3C). The observation of habitat-specific clusters in the ordination is also present in the individual hierarchical clustering approaches (Figs. S2B–S2D). In addition, for seawater (habitat-based PERMANOVA) the relative abundance, and the sponge & seawater (group-based permanova) presence-absence groups the significant multivariate spread (Dispersion) might contribute to the observed significant variance effects (Table 3).

**Figure 4:**
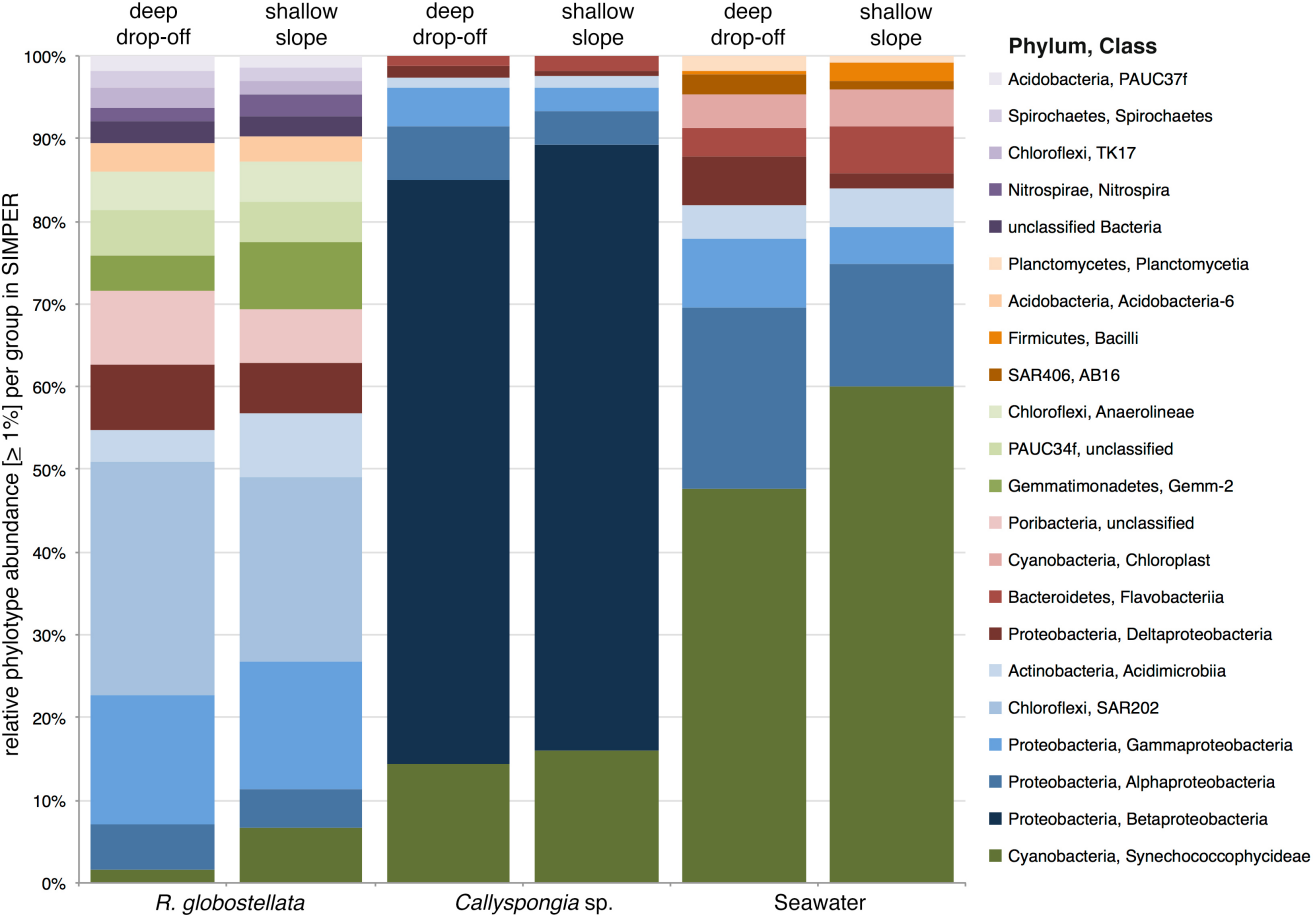
SIMPER contributions to Bray-Curtis dissimilarities between habitats. Mean sequence abundances of the 97%-OTUs which contribute the most to overall Bray-Curtis dissimilarities, as calculated by SIMPER among open and deep *R. globostellata*, *Callyspongia* sp., and seawater samples. Individual 97%-OTUs were collapsed at class level and only considered with an overall abundance of >1%.

**Figure 5:**
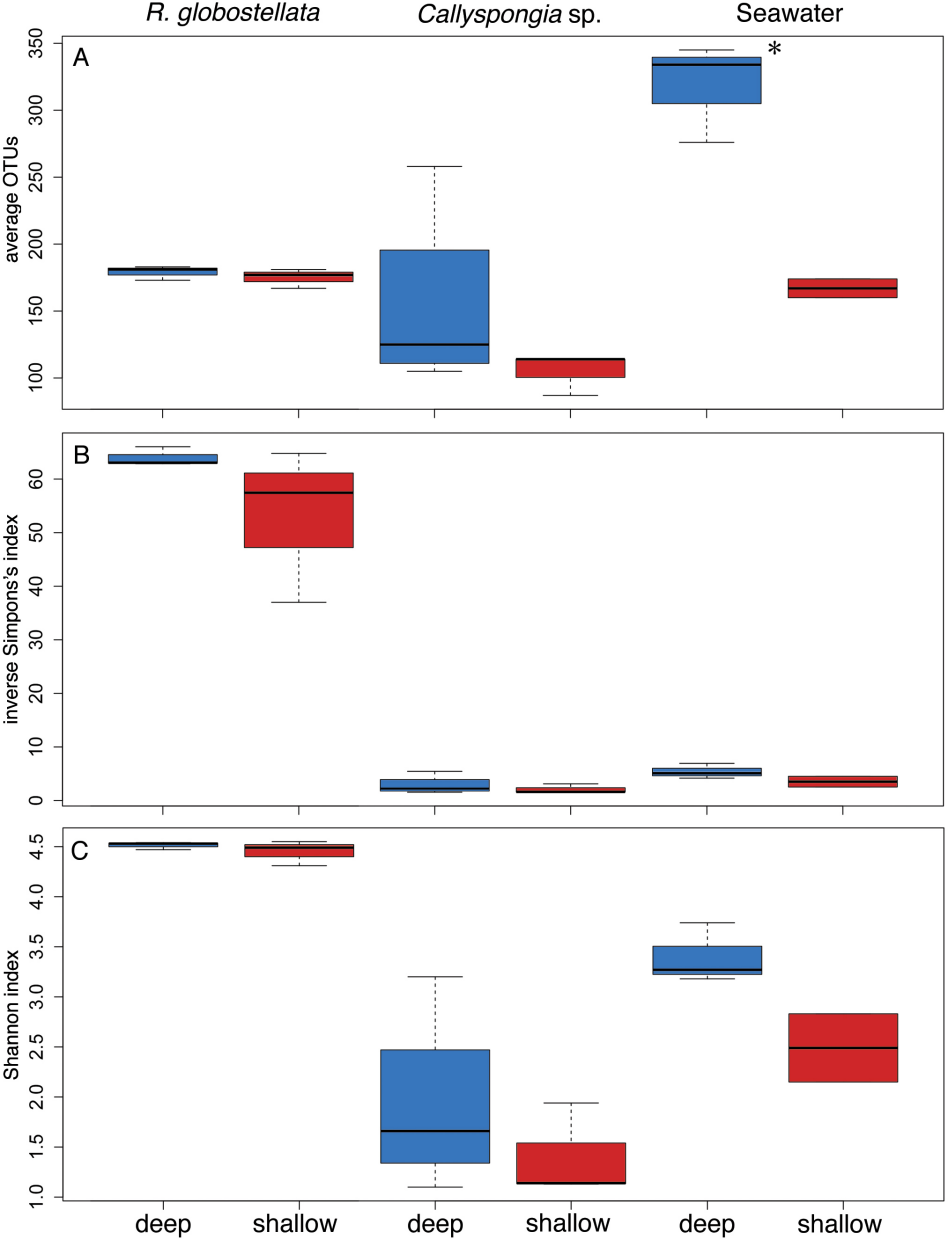
Alpha diversity comparisons between habitats. Number of (A) average 97%-OTUs and alpha diversity estimates, (B) inverse Simpson’s and (C) Shannon index for shallow reef slopes and deep reef drop-off *R. globostellata*, *Callyspongia* sp. and seawater samples. Top, middle, and bottom lines of the boxes represent the 25th, 50th (median), and 75th percentiles, respectively. The end of the whiskers represent the 5th and 95th percentiles, respectively. Blue and red habitat colors correspond with the color code in Fig. 3. Measurements of observed average OTUs, inverse Simpson’s index and Shannon index were analyzed using a one-way analysis of variance (ANOVA, *p* < 0.05) using habitat as a fixed factor. Significant results are marked by an asterisk.

The graphical summary of the OTUs (collapsed to high taxonomic ranks) with the highest average abundance in SIMPER in each of the sample groups presents three distinct microbial communities, with only slight variations between the two habitats (Fig. 4). For example, *R. globostellata* harbours an abundant Chloroflexi community and is the only sample group containing the candidate phylum “Poribacteria.” In contrast, *Callyspongia* sp. was dominated by members of Betaproteobacteria and seawater samples by Cyanobacteria and Gammaproteobacteria. In all three sample types, Cyanobacteria were slightly more abundant in samples from the shallow reef slope habitat. In contrast to the overall balanced taxonomic contribution patterns between habitats at higher taxonomic ranks, the analysis of 97%-OTUs showed individual habitat contributions and that several OTUs with a high abundance among all samples were also main contributors to the overall dissimilarity among the shallow and very deep sample groups (Table 4). The most prominent feature among all three analyzed sample groups was the dominance of *Synechococcus* (phylum Cyanobacteria) in the shallow reef slope habitats. In the deep drop-off *Callyspongia* sp. and seawater samples a second cyanobacterium, genus *Prochlorococcus* (OTU0003), was most dominant. The dominant *Synechococcus* OTUs were also separated in *R. globostellata* (OTU0016) and *Callyspongia* sp. and seawater (OTU0002, OTU0037, OTU0041). Additionally, compared to *Callyspongia* sp. and seawater, which exhibit dominant OTUs either in shallow reef slopes or deep habitats, the main contributing OTUs in *R. globostellata* dominate completely the specimens from shallow habitat (Table 4). The contrasting and habitat-dependent patterns were also prominent in the average species richness and the two alpha diversity indices among the three groups (Figs. 5A–5C); *R. globostellata* represents the sponge with the highest evenness, richness and OTU dominance compared to *Callyspongia* sp. and seawater samples. Additionally, all three sample groups exhibit higher richness and diversity values in the deep drop-off habitats and individual variations among the shallow reef slopes and deep drop-off habitats.

**Table 4:** Most abundant SIMPER OTUs. Average abundance and cumulative contribution of the top 15 dominant 97%-OTUs contributing at least to 70% of the Bray-Curtis dissimilarities (SIMPER). Calculated among shallow reef slopes and deep reef drop-off *R. globostellata*, *Callyspongia* sp. and seawater samples. Microbial taxonomy is based on the Greengenes 97%-OTU classification from phylum to species level (if applicable). Habitat-specific OTU prevalence emphasized in bold; cusum: ordered cumulative contribution.

		Average abundance			
	OTUs	Deep	Shallow	Cusum	Microbial taxonomy
*R. globostellata*	Otu0016	45.67	**534.67**	0.09	Cyanobacteria, Synechococcophycideae, Synechococcales, Synechococcaceae, *Synechococcus*
	Otu0006	109.33	**529.33**	0.17	Actinobacteria, Acidimicrobiia, Acidimicrobiales, wb1_P06
	Otu0017	72.67	**216.00**	0.19	Nitrospirae, Nitrospira, Nitrospirales, Nitrospiraceae
	Otu0072	1.33	**146.33**	0.22	Gemmatimonadetes, Gemm-2
	Otu0034	42.33	**184.33**	0.24	Proteobacteria, Gammaproteobacteria, Thiotrichales, Piscirickettsiaceae
	Otu0054	0.67	**107.00**	0.26	Poribacteria
	Otu0042	21.67	**125.33**	0.28	Proteobacteria, Gammaproteobacteria, Thiotrichales, Piscirickettsiaceae
	Otu0020	106.00	**195.33**	0.30	Chloroflexi, SAR202
	Otu0089	6.00	**105.33**	0.32	Bacteroidetes, Rhodothermi, Rhodothermales, Rhodothermaceae, Salisaeta
	Otu0013	91.00	**182.00**	0.34	Proteobacteria, Gammaproteobacteria, Chromatiales, Ectothiorhodospiraceae
	Otu0046	38.00	**126.00**	0.35	Gemmatimonadetes, Gemm-2
	Otu0021	66.33	**151.33**	0.37	PAUC34f
	Otu0008	199.67	**242.33**	0.38	Poribacteria
	Otu0093	11.00	**95.00**	0.40	Gemmatimonadetes, Gemm-2
	Otu0012	53.00	**127.33**	0.41	Proteobacteria, Gammaproteobacteria
*Callyspongia* sp.	Otu0001	3147.25	**3153.67**	0.41	Proteobacteria, Betaproteobacteria, EC94
	Otu0002	52.75	**628.67**	0.57	Cyanobacteria, Synechococcophycideae, Synechococcales, Synechococcaceae, *Synechococcus*
	Otu0003	**585.00**	21.33	0.71	Cyanobacteria, Synechococcophycideae, Synechococcales, Synechococcaceae, *Prochlorococcus*
	Otu0005	**117.00**	68.67	0.72	Proteobacteria, Alphaproteobacteria, Rickettsiales, Pelagibacteraceae
	Otu0073	37.25	**38.00**	0.73	Proteobacteria
	Otu0152	**35.75**	10.33	0.74	Proteobacteria, Gammaproteobacteria, Oceanospirillales, Endozoicimonaceae
	Otu0037	1.00	**24.00**	0.75	Cyanobacteria, Synechococcophycideae, Synechococcales, Synechococcaceae, *Synechococcus*
	Otu0011	31.25	**49.00**	0.75	Actinobacteria, Acidimicrobiia, Acidimicrobiales, OCS155
	Otu0041	0.75	**19.33**	0.76	Cyanobacteria, Synechococcophycideae, Synechococcales, Synechococcaceae, *Synechococcus*
	Otu0025	7.00	**24.00**	0.76	Proteobacteria, Alphaproteobacteria, Rhodobacterales, Rhodobacteraceae
	Otu0232	7.25	**13.67**	0.77	Proteobacteria, Gammaproteobacteria, Oceanospirillales, Endozoicimonaceae
	Otu0044	8.00	**21.33**	0.77	Proteobacteria, Alphaproteobacteria, Rhodobacterales, Rhodobacteraceae
	Otu0284	**12.50**	1.33	0.77	Proteobacteria, Gammaproteobacteria, Alteromonadales, Shewanellaceae, *Shewanella*
	Otu0059	10.50	**20.33**	0.78	Bacteroidetes, Flavobacteriia, Flavobacteriales, Flavobacteriaceae
	Otu0157	**13.75**	2.33	0.78	Planctomycetes, Planctomycetia, Pirellulales, Pirellulaceae
Seawater	Otu0002	314.00	**7548.00**	0.34	Cyanobacteria, Synechococcophycideae, Synechococcales, Synechococcaceae, *Synechococcus*
	Otu0003	**5147.67**	60.00	0.58	Cyanobacteria, Synechococcophycideae, Synechococcales, Synechococcaceae, *Prochlorococcus*
	Otu0005	**711.33**	630.50	0.60	Proteobacteria, Alphaproteobacteria, Rickettsiales, Pelagibacteraceae
	Otu0037	3.00	**432.50**	0.62	Cyanobacteria, Synechococcophycideae, Synechococcales, Synechococcaceae, *Synechococcus*
	Otu0011	398.33	**628.50**	0.64	Actinobacteria, Acidimicrobiia, Acidimicrobiales, OCS155
	Otu0041	5.00	**393.00**	0.66	Cyanobacteria, Synechococcophycideae, Synechococcales, Synechococcaceae, *Synechococcus*
	Otu0025	96.67	**462.00**	0.67	Proteobacteria, Alphaproteobacteria, Rhodobacterales, Rhodobacteraceae
	Otu0058	4.33	**328.00**	0.69	Firmicutes, Bacilli, Bacillales, Bacillaceae, Bacillus
	Otu0071	32.67	**234.50**	0.70	Cyanobacteria, Chloroplast, Chlorophyta, Mamiellaceae
	Otu0061	**198.33**	18.50	0.70	Proteobacteria, Deltaproteobacteria, Sva0853, SAR324
	Otu0032	**257.33**	111.00	0.71	Proteobacteria, Alphaproteobacteria, Rickettsiales, Pelagibacteraceae
	Otu0044	71.67	**244.00**	0.72	Proteobacteria, Alphaproteobacteria, Rhodobacterales, Rhodobacteraceae
	Otu0076	**177.67**	11.50	0.73	Proteobacteria, Alphaproteobacteria, Sphingomonadales, Erythrobacteraceae, *Erythrobacter*
	Otu0080	**131.67**	35.00	0.73	Proteobacteria, Alphaproteobacteria, Rhodobacterales, Rhodobacteraceae
	Otu0148	4.67	**102.00**	0.74	Planctomycetes, OM190, CL500-15

## Discussion

### Host specificity of the microbiota of four MCE sponges

In the present study divergent patterns between two different habitats were visible in the tropical sponge and seawater microbiota. *Callyspongia* sp. and seawater samples appear to have an intrinsic microbial community composition, which is variable enough to significantly separate the intra-species communities by their shallow reef slope or deep drop-off habitats. On the other hand *R. globostellata* exhibits an observable difference in community variation that is not significant. In addition, we could also observe significant microbial specificity across all analyzed sponge taxa independent of habitat. Since the shallow collection sites for *Callyspongia* sp. and *R. globostellata* inside Apra Harbor represent the same shallow water fore reef slope habitat dominated by *Porites rus* corals, it is unlikely that the observed differences in beta diversity between shallow reef slope or deep drop-off habitats are due to collection of *Callyspongia* sp. from the two shallow Apra Harbor sites.

Little is known about the *R. globostellata* and *Rhaphoxya* sp. microbial communities. Culture-dependent approaches reported only Actinobacteria, Bacteroidetes, Firmicutes, Proteobacteria (Alpha & Gamma) from *R. globostellata* (Lafi, Garson & Fuerst, 2005; Steinert et al., 2014). In addition, Lafi et al. (2009) found the candidate phylum “Poribacteria” in this demosponge. While the first high-throughput-sequencing amplicon screening detected 16 bacterial phyla associate with *R. globostellata* (Schmitt et al., 2012), we can now increase the number to a total of 23 microbial phyla. For *Rhaphoxya* sp., virtually nothing has been known about the associated microbiota. However, this sponge taxon is already the focus of natural products research, which hints at a chemically active symbiotic microbiota (Wright et al., 2012). Here we present phylum and OTU composition patterns in *R. globostellata* and *Rhaphoxya* sp. that are surprisingly similar. While designation of the two sponge taxa as either high microbial abundance (HMA) or low microbial abundance (LMA) sponges is lacking in the literature, the associated microbial phylotypes are congruent with the microbial phyla commonly found in association with HMA sponges (i.e., Proteobacteria, Chloroflexi, Acidobacteria, Actinobacteria, and the candidate phylum “Poribacteria”) (Schmitt et al., 2011; Gloeckner et al., 2014).

Recent transmission electron microscopy, DAPI cell-counting and 16S rRNA gene amplicon sequencing results assigned different members of the demosponges *A*. *cavernosa* and *Callyspongia* sp. to the LMA group, with abundant Proteobacteria (Alpha, Beta, & Gamma) and Cyanobacteria (*Synechococcus*) microbial community members (Gloeckner et al., 2014; Giles et al., 2013; Jeong, Kim & Park, 2013). We also found that our *Callyspongia* sp. specimens exhibited an abundant occurrence of *Synechococcus* (Cyanobacteria), Actinobacteria, Bacteroidetes and Proteobacteria (Beta & Gamma). Within the Betaproteobacteria, the order EC94 exhibited the highest overall abundance in our *Callyspongia* sp. samples and among all OTUs. Reports on this order are rare, but recent community analyses on different demosponges found this microbial taxon to be dominant in the deep sea sponge *Inflatella pellicula* and several shallow water sponges from Korea (Jeong, Kim & Park, 2015; Jackson et al., 2013; Jeong, Kim & Park, 2013).

Overall, sponge and seawater community structures correlated significantly with host identity. This underlines the common view that host identity is an important factor for the composition of sponge-associated microbial communities (e.g., Cárdenas et al., 2014; Cleary et al., 2013; Easson & Thacker, 2014; Pita et al., 2013). Recent studies on several sponge species showed that the microbiota of LMA sponges, in particular, exhibits a low degree of similarity among the investigated sponge species (Giles et al., 2013; Blanquer, Uriz & Galand, 2013). The sponge genera *Acanthella* and *Callyspongia* were previously found to include LMA sponges (Gloeckner et al., 2014), and the bacterial community ordination, phylum composition and OTU structures of the present study separates these two sponge taxa very distinctly from the other two investigated sponges (*Rhabdastrella, Rhaphoxya*). In addition, the observed microbial community patterns in *Callyspongia* sp. appear to be more closely related to the seawater samples (see OTU and phylum composition). This high similarity with seawater communities is a well-known feature of LMA sponges (e.g., Taylor et al., 2007; Schmitt et al., 2011; Blanquer, Uriz & Galand, 2013). In contrast, HMA members are commonly more closely related to each other, especially after the removal of potential environmental sources of variation (Blanquer, Uriz & Galand, 2013). Finally, four sponge samples (two *A. cavernosa* and two *Callyspongia* sp.) have been sampled at different points in time. However, a temporal effect on the community structure is not evident, which is in accordance with recent research showing the low seasonal variability of the sponge-microbiota (Erwin et al., 2012; Erwin et al., 2015), although other similar studies have indicated the temporal variability of sponge-associated bacteria (White et al., 2012; Anderson, Northcote & Page, 2010).

### Depth-dependent microbial community patterns in MCE sponges

Knowledge about variability of the sponge microbiota along environmental gradients on local spatial scales is still scarce (Olson & Kellogg, 2010). A T-RFLP and clone library study on three MCE sponges identified a trend in community composition along a depth gradient, but could not identify the bacteria which caused these variations (Olson & Gao, 2013). Correspondingly, a recent 16S rRNA gene amplicon analysis found significant shifts in the *X. muta*-associated microbial community along a depth gradient from 10 to 90 m and demonstrated that environmental factors may influence the sponge microbiota (Morrow, Fiore & Lesser, 2016). A sponge transplantation experiment showed little overall effect between different habitats (Cárdenas et al., 2014), and comparisons between sponges obtained from different habitats (marine lake vs. coastal system & intertidal vs. subtidal) showed the importance of host relatedness and habitat as determinants of microbial community structure (Weigel & Erwin, 2015; Cleary et al., 2013).

In the present study, *Callyspongia* sp. and seawater microbial communities were significantly different when comparing shallow reef slope and deep drop-off habitats. In contrast, based on the multivariate statistical tests, *R. globostellata* microbial communities were not significantly affected by the different habitats (shallow versus deep), which leads to the conclusion that in this particular case the observed habitat-specific nMDS and hierarchical clustering patterns are random variations of the microbial community composition. In order to sample in two environmentally very distinct but closely related habitats, we focused sampling on “shallow” and “very deep” depths as categorized by Brazeau, Lesser & Slattery (2013). Irradiance, but also nutrient availability and water temperature, are dependent on both depth and changing environmental factors along spatial gradients from shallow to mesophotic coral reefs (see Lesser, Slattery & Leichter, 2009; Olson & Gao, 2013). The observed temperature difference between shallow reef slope and deep drop-off sites averaged 4 °C, similar to that reported by Lesser et al. (2010); difference of 4 °C between 3 m and 91 m depth). While temperature is one indication for environmental differences between the two habitats, it seems unlikely that the observed difference of 4 °C will affect sponge-microbe communities between the two habitats to the extent reported in this study. Studies examining the effect of elevated temperatures found no change (at sub-lethal temperatures) in sponge bacterial communities during short-term experiments (Webster, Cobb & Negri, 2008; Simister et al., 2012b). It is likely that other local environmental factors such as light have a considerable effect on the small-scale patterns observed here, especially as similar studies on mesophotic reefs by Lesser, Slattery & Leichter (2009) and Lesser et al. (2010) did observe pronounced differences in light along similar depth gradients.

Within *Callyspongia* sp. and seawater samples, either Cyanobacteria or Proteobacteria OTUs were mainly responsible for the differences observed by SIMPER. In particular, cyanobacterial *Synechococcus* OTUs were among the main contributors to the shallow microbiota, whereas only one *Prochlorococcus* OTU was a dominant cyanobacterium contributing to the deep group from mesophotic depths. The predominance of photoautotrophs in tropical filter-feeding sponges is intuitive given the widespread prevalence of cyanobacteria in the ocean. LMA sponges in particular are known to harbour cyanobacteria in high abundance (Bayer, Kamke & Hentschel, 2014), and cyanobacteria comprise one of the most abundant sponge-associated phyla, with well-established sponge-specific symbionts (e.g., *Synechococcus spongarium*) (Hentschel, Usher & Taylor, 2006; Taylor et al., 2007; Simister et al., 2012a). Moreover, LMA sponges exhibit higher water filtering capabilities, presumably due to a less dense mesohyl and less complex aquiferous system compared to HMA sponges (Weisz, Lindquist & Martens, 2008). Therefore, the congruent microbial community patterns between *Callyspongia* sp. and seawater samples observed here, in contrast to those of *R. globostellata*, could be correlated with physiological differences between HMA and LMA sponges (see Weisz, Lindquist & Martens, 2008; Thacker & Freeman, 2012). Besides the potential phototrophic activities in the sponge pinacoderm (outer tissue) and mesohyl (inner sponge matrix), nitrogen-fixing cyanobacteria may inhabit a niche within the complex nitrogen cycle in sponges, with a mutual benefit due to nutrient supply by the sponge and secondary metabolite production by the cyanobacteria (Taylor et al., 2007; Wilkinson & Fay, 1979; Arillo et al., 1993). Given the dominance of Cyanobacteria in our *Callyspongia* sp. specimens and seawater samples, with *Synechococcus* dominant in the shallow and *Prochlorococcus* in the deep sponges, the spatial pattern could be shaped predominantly by cyanobacterial lineages. This distribution pattern of microbes in *Callyspongia* sp. could also indicate a seasonal vertical distribution pattern, in which one genus dominates the shallow high-light water column, while the other genus is temporarily mainly present in the low-light area below. While temporal shifts of Cyanobacteria in sponges have been observed to varying degrees previously, the combined effect of time and depth on these and other chlorophototrophs in sponges remains uncertain (White et al., 2012; Erwin et al., 2012; Hardoim & Costa, 2014; Taylor et al., 2004). A recent analysis on stable isotopes indicated that, with increasing depth, the inorganic nutrients dependency in sponges shifts from photoautotrophy to heterotrophy, in accordance with a significant shift in the associated microbiota (Morrow, Fiore & Lesser, 2016). Conversely, in the same sponge species at a different location an observed stable isotopic enrichment correlated with a larger microbial community similarity across different sampling depths. In addition, other factors should be considered to explain the observed differences. Since the same sponge species show differences in growth rates and species richness at different depths (Lesser, Slattery & Leichter, 2009), different biotic and abiotic niches may be available for symbionts depending on their habitat. Such ecologically-based niche differentiation for symbionts and hosts is known for the tropical corals *Seriatopora hystrix* (Bongaerts et al., 2010) and *Montastraea cavernosa* (Lesser et al., 2010; Brazeau, Lesser & Slattery, 2013). Recent oligotyping of Nitrospira symbionts associated with sponges collected along large horizontal and vertical gradients provided further evidence for such patterns of differential enrichment of closely related microbial variants (Reveillaud et al., 2014).

In *R. globostellata*, one cyanobacterial OTU (OTU0016) is predominant within the deep sheltered communities, but also present at high abundance in the shallow specimens of this species (Table 4). Moreover, it was also largely absent from all other sponge and seawater samples. The BLAST search against the NCBI nucleotide collection revealed that this particular OTU is highly similar to *Candidatus* Synechococcus spongiarum, a symbiotic cyanobacterium found in many sponges (Hentschel, Usher & Taylor, 2006). Compared to this, the main cyanobacterial OTUs found in *Callyspongia* sp. and seawater (OTU0002 & OTU0003) yielded different BLAST results (uncultured *Synechococcus* sp. clone & *Prochlorococcus* sp., respectively). This corresponds to the theory that LMA sponges generally acquire their microbial symbionts via horizontal transmission from the surrounding environment, while HMA sponges possess a more individual microbial community that does not mirror the surrounding seawater microbiome as closely as their low abundance counterparts (Gloeckner et al., 2014; Hentschel, Usher & Taylor, 2006). The detection of microbes within different reproductive stages of seven sponge species led to the hypothesis that HMA sponges can maintain parts of their symbiotic microbiota via vertical transmission (Schmitt et al., 2008). Interestingly, the major microbial drivers contributing to the observed differences in beta diversity are all located in the deep sponges. Given that the abundant microbial phyla in *R. globostellata* (i.e., Acidobacteria, Chloroflexi, Cyanobacteria, Gemmatimonadetes, and Alphaproteobacteria) are known to contain (bacterio)chlorophyll-based phototrophic lineages (Zeng et al., 2014), it is possible that photoheterotrophic bacteria also play a considerable role in this host-specific microbiota. However, while the function of Chloroflexi in sponges is yet unclear, the distribution of members of this phylum within their hosts from different depths suggests that they may not be phototrophically active within the sponges (Olson & Gao, 2013).

## Conclusion

The present study suggests that sponge-specific communities in tropical coral ecosystems are predominantly influenced by host identity. Moreover, the variance between *Callyspongia*-associated microbial communities from two different habitats (i.e., shallow reef slopes and deep drop-off reefs) is large enough to observe significant differences. However, the actual environmental factors contributing to the observed habitat-dependent variances remain uncertain, although we speculate that temperature may be less likely to have caused the variations in the sponge microbiota between shallow and deep specimens. While sponge-microbe communities show an overall stability along large geographic and temporal gradients, local environmental factors may have an effect on the small-scale patterns observed here. To further test the hypothesis of differential sponge-associated microbial communities along local depth gradients, functional and temporal aspects should be considered in future *in situ* studies. Moreover, since temporal turnover of phytoplankton is faster in the tropics (Soininen, 2010), tropical sponges with dominant phototrophic microbial communities most likely provide ideal conditions to design spatio-temporal studies on phototrophic host-symbiont dynamics.

## Supplemental Information

10.7717/peerj.1936/supp-1 Figure S1 Rarefaction curves Rarefied 97%-OTU 16S rRNA gene amplicon data for each sample.

10.7717/peerj.1936/supp-2 Figure S2 Hierarchical OTU clustering Dendrograms showing the Bray-Curtis dissimilarity of microbial communities of (A) the complete sample dataset, (B) *Callyspongia* sp., (C) *R. globostellata*, (D) seawater, (E) *A. cavernosa*, (F) *Rhaphoxya* sp. sponge specimens based on 97%-OTU amplicon subsets.

10.7717/peerj.1936/supp-3 Table S1 Mapping data Habitat file used for grouping of samples in multivariate analyses (nMDS & adonis).

10.7717/peerj.1936/supp-4 Table S2 Tukey multiple betadisper comparisons Tukey multiple comparisons of means for the group based betadisper analysis. 95% family-wise confidence level—diff giving the difference in the observed means, lwr giving the lower end point of the interval, upr giving the upper end point and *p* adj giving the *p*-value after adjustment for the multiple comparisons.

10.7717/peerj.1936/supp-5 Table S3 Betadisper pairwise comparisons Pairwise comparisons of the group based permutation test for homogeneity of multivariate dispersions. Observed *p*-value below diagonal, permuted *p*-value above diagonal.

## Additional Information and Declarations

### DNA Deposition

The following information was supplied regarding the deposition of DNA sequences: The obtained raw sequence data can be accessed via the NCBI Sequence Read Archive under accession number SRX838037.

### Data Availability

The following information was supplied regarding data availability: The 97% OTU abundance table combined with the Greengenes classification for each individual OTU can be accessed via the Figshare online repository— 10.6084/m9.figshare.2063280. The best hits and representative sequences for the most abundant OTUs can be accessed via the Figshare online repository (10.6084/m9.figshare.2366827).
