# Supplementary figures and images for: In four shallow and mesophotic tropical reef sponges from Guam the microbial community largely depends on host identity
Source: PeerJ. 2016 Apr 18;4:e1936. doi: 10.7717/peerj.1936 (PMC4841226; doi:10.7717/peerj.1936)

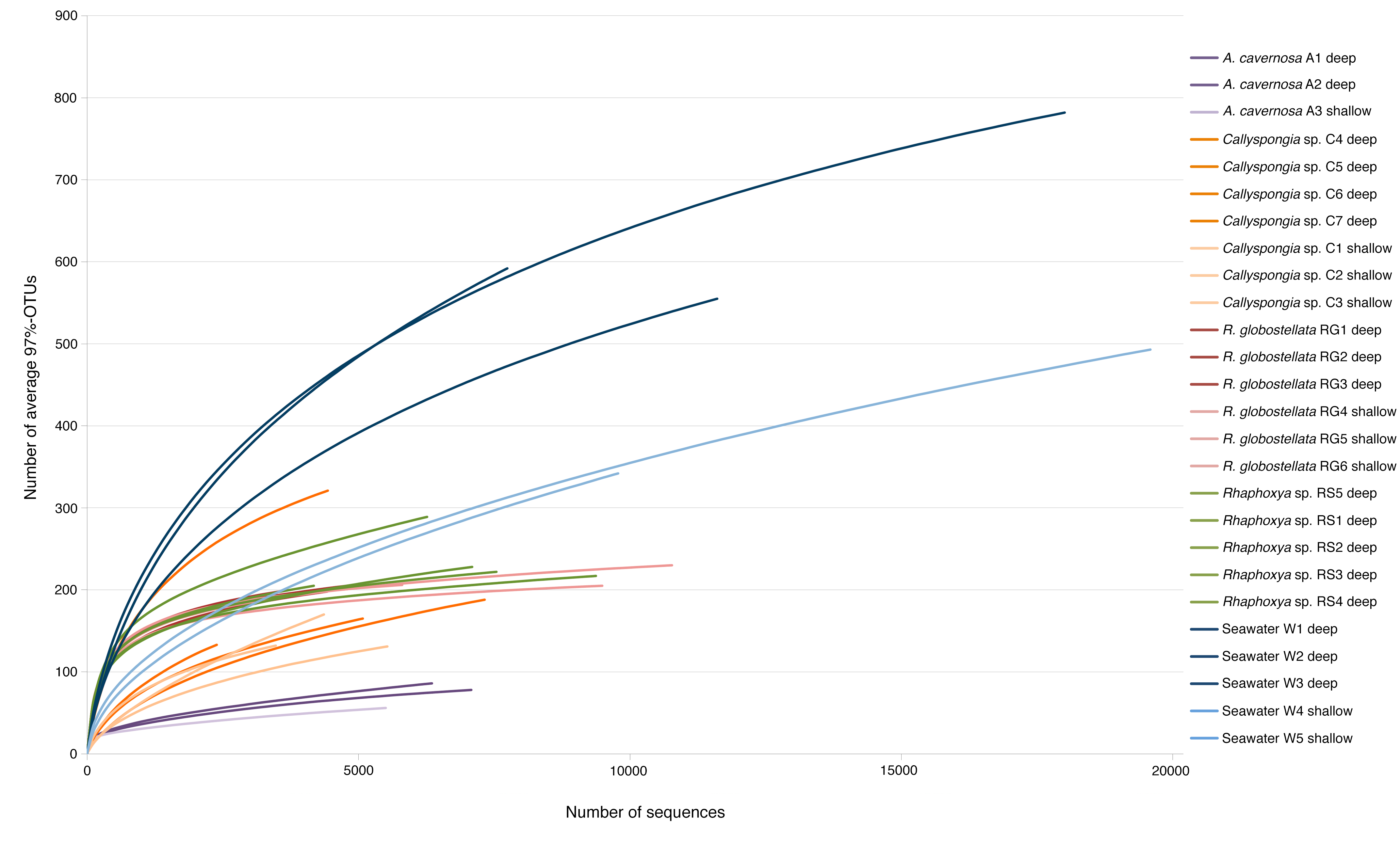

Supplement: Figure S1 — Rarefied 97%-OTU 16S rRNA gene amplicon data for each sample. [file peerj-04-1936-s001.png]

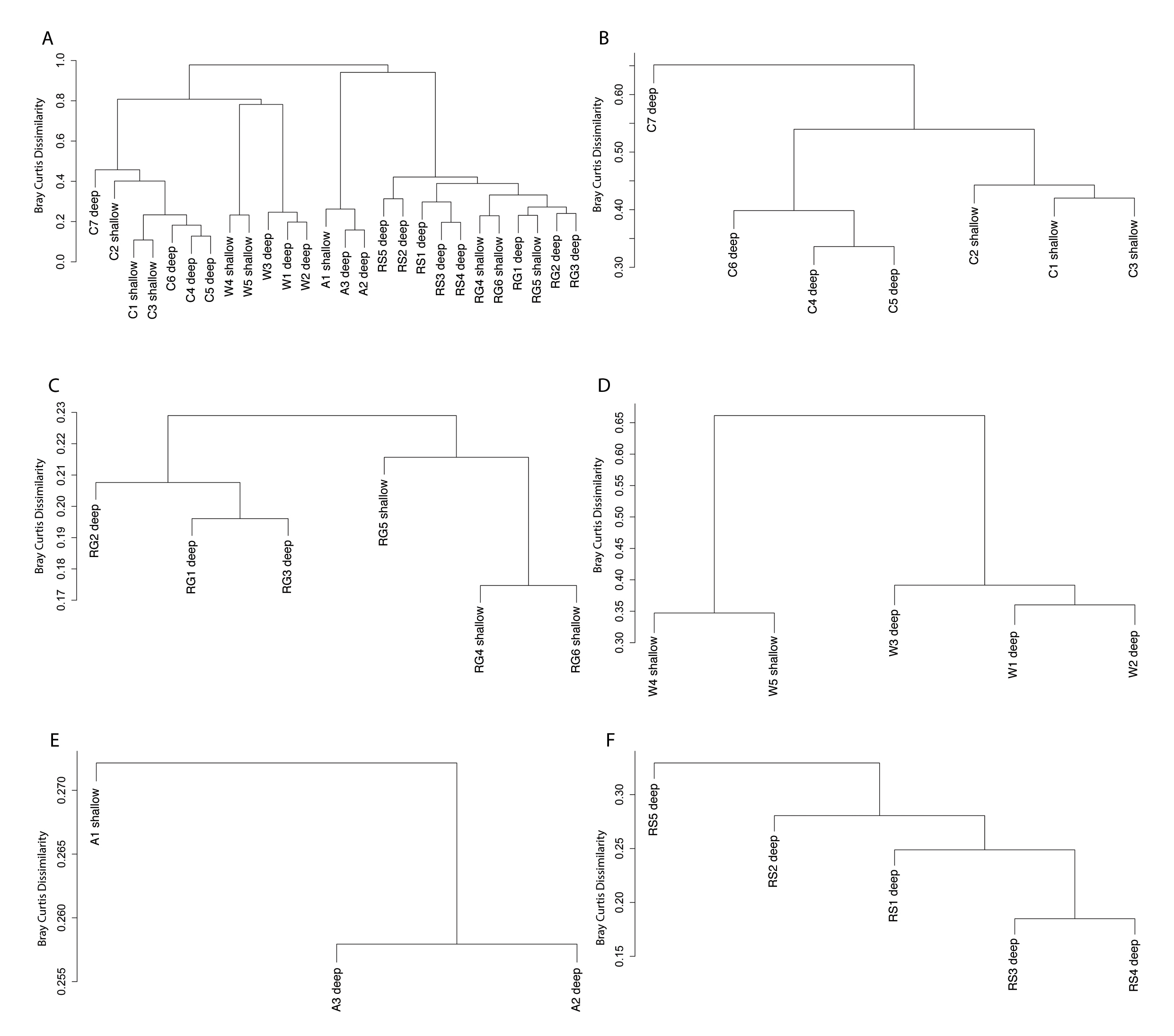

Supplement: Figure S2 — Dendrograms showing the Bray-Curtis dissimilarity of microbial communities of (A) the complete sample dataset, (B) Callyspongia sp., (C) R. globostellata, (D) seawater, (E) A. cavernosa, (F) Rhaphoxya sp. sponge specimens based on 97%-OTU amplicon subsets. [file peerj-04-1936-s002.png]
