# Supplementary material for: In four shallow and mesophotic tropical reef sponges from Guam the microbial community largely depends on host identity
Source: PeerJ. 2016 Apr 18;4:e1936. doi: 10.7717/peerj.1936 (PMC4841226; doi:10.7717/peerj.1936)
Supplement: Table S1 — Habitat file used for grouping of samples in multivariate analyses (nMDS & adonis). [file peerj-04-1936-s003.docx]

| accession | name | habitat | group | MID GeoB.sff | MID MikeH.sff |
| --- | --- | --- | --- | --- | --- |
| Acan_shallow_PS701 | A1 | shallow slope | Acanthella | TAGAGACGAG |  |
| Acan_deep_PS773 | A2 | deep drop-off | Acanthella | ACGCGAGTAT |  |
| Acan_deep_PS508 | A3 | deep drop-off | Acanthella | ACTACTATGT |  |
| Cally_shallow_PSC1 | C1 | shallow slope | Callyspongia |  | TCTCTATGCG |
| Cally_shallow_PSC2 | C2 | shallow slope | Callyspongia | TGATACGTCT |  |
| Cally_shallow_PSC3 | C3 | shallow slope | Callyspongia |  | TGATACGTCT |
| Cally_deep_PS483 | C4 | deep drop-off | Callyspongia | CGAGAGATAC |  |
| Cally_deep_PS484 | C5 | deep drop-off | Callyspongia |  | TAGTATCAGC |
| Cally_deep_PS700 | C6 | deep drop-off | Callyspongia | TACACACACT |  |
| Cally_deep_PS719 | C7 | deep drop-off | Callyspongia | TACACGTGAT |  |
| Rhabda_deep_PS1 | RG1 | deep drop-off | Rhabdastrella | ACATACGCGT |  |
| Rhabda_deep_PS2 | RG2 | deep drop-off | Rhabdastrella | ACTGTACAGT |  |
| Rhabda_deep_PS4 | RG3 | deep drop-off | Rhabdastrella | AGACTATACT |  |
| Rhabda_shallow_PS1 | RG4 | shallow slope | Rhabdastrella | AGCGTCGTCT |  |
| Rhabda_shallow_PS2 | RG5 | shallow slope | Rhabdastrella | AGTACGCTAT |  |
| Rhabda_shallow_PS3 | RG6 | shallow slope | Rhabdastrella | ATAGAGTACT |  |
| Rhaphox_deep_PS718 | RS1 | deep drop-off | Rhaphoxya |  | CTCGCGTGTC |
| Rhaphox_deep_PS720 | RS2 | deep drop-off | Rhaphoxya | ACGAGTGCGT |  |
| Rhaphox_deep_PS723 | RS3 | deep drop-off | Rhaphoxya | ACGCTCGACA |  |
| Rhaphox_deep_PS724 | RS4 | deep drop-off | Rhaphoxya | ATCAGACACG |  |
| Rhaphox_deep_PS699 | RS5 | deep drop-off | Rhaphoxya | TCTCTATGCG |  |
| water_deep1 | W1 | deep drop-off | watercolumn |  | ACGCTCGACA |
| water_deep2 | W2 | deep drop-off | watercolumn |  | AGACGCACTC |
| water_deep3 | W3 | deep drop-off | watercolumn |  | AGCACTGTAG |
| water_shallow1 | W4 | shallow slope | watercolumn |  | ATCAGACACG |
| water_shallow2 | W5 | shallow slope | watercolumn |  | ATATCGCGAG |
