# Supplementary material for: In four shallow and mesophotic tropical reef sponges from Guam the microbial community largely depends on host identity
Source: PeerJ. 2016 Apr 18;4:e1936. doi: 10.7717/peerj.1936 (PMC4841226; doi:10.7717/peerj.1936)
Supplement: Table S2 — Tukey multiple comparisons of means for the group based betadisper analysis. 95% family-wise confidence level—diff giving the difference in the observed means, lwr giving the lower end point of the interval, upr giving the upper end point and p adj giving the p-value after adjustment for the multiple comparisons. [file peerj-04-1936-s004.docx]

| Group | diff | lwr | upr | *p* adj |
| --- | --- | --- | --- | --- |
| *Callyspongia-Acanthella* | 0.1940 | 0.0523 | 0.3356 | **0.0044** |
| *Rhabdastrella-Acanthella* | -0.0127 | -0.1579 | 0.1324 | 0.9989 |
| *Rhaphoxya-Acanthella* | 0.0235 | -0.1264 | 0.1735 | 0.9895 |
| watercolumn-*Acanthella* | 0.1974 | 0.0475 | 0.3474 | **0.0063** |
| *Rhabdastrella-Callyspongia* | -0.2067 | -0.3209 | -0.0925 | **0.0002** |
| *Rhaphoxya-Callyspongia* | -0.1704 | -0.2906 | -0.0502 | **0.0031** |
| watercolumn-*Callyspongia* | 0.0035 | -0.1167 | 0.1237 | 1.0000 |
| *Rhaphoxya-Rhabdastrella* | 0.0363 | -0.0881 | 0.1606 | 0.9051 |
| watercolumn-*Rhabdastrella* | 0.2102 | 0.0859 | 0.3345 | **0.0005** |
| watercolumn-*Rhaphoxya* | 0.1739 | 0.0441 | 0.3037 | **0.0054** |
