# Supplementary material for: In four shallow and mesophotic tropical reef sponges from Guam the microbial community largely depends on host identity
Source: PeerJ. 2016 Apr 18;4:e1936. doi: 10.7717/peerj.1936 (PMC4841226; doi:10.7717/peerj.1936)
Supplement: Table S3 — Pairwise comparisons of the group based permutation test for homogeneity of multivariate dispersions. Observed p-value below diagonal, permuted p-value above diagonal. [file peerj-04-1936-s005.docx]

| Source | *Acanthella* | *Callyspongia* | *Rhabdastrella* | *Rhaphoxya* | watercolumn |
| --- | --- | --- | --- | --- | --- |
| *Acanthella* | - | **0.0070** | 0.1270 | 0.5320 | **0.0210** |
| *Callyspongia* | **0.0057** | - | **0.0010** | **0.0020** | 0.9620 |
| *Rhabdastrella* | 0.1417 | **0.0001** | - | 0.1520 | **0.0030** |
| *Rhaphoxya* | 0.5182 | **0.0033** | 0.1489 | - | **0.0070** |
| watercolumn | **0.0179** | 0.9502 | **0.0007** | **0.0102** | - |
